# Effect of coffee consumption on fetal renal artery blood flow and amniotic fluid volume in third trimester of pregnancy

**DOI:** 10.12669/pjms.36.4.1690

**Published:** 2020

**Authors:** Ilknur Col Madendag, Mefkure Eraslan Sahin, Emine Aydin, Yusuf Madendag

**Affiliations:** 1 Ilknur Col Madendag, MD. Department of Obstetrics and Gynecology, Health Sciences University, Kayseri City Hospital, Kayseri, Turkey; 2 Mefkure Eraslan Sahin, MD. Department of Obstetrics and Gynecology, Health Sciences University, Kayseri City Hospital, Kayseri, Turkey; 3 Emine Aydin, MD. Department of Perinatology, Health Sciences University, Kayseri City Hospital, Kayseri, Turkey; 4 Yusuf Madendag, MD. Department of Obstetrics and Gynecology, Erciyes University, Faculty of Medicine, Kayseri, Turkey

**Keywords:** Amniotic fluid index, Caffeine, Coffee, Oligohydramnios

## Abstract

**Objective:**

Coffee is frequently (one or two cups/day) consumed throughout pregnancy. Although there are a few studies evaluating caffeine effects on pregnancy; however, a diuretic effect of caffeine on fetal kidneys has not been reported. Therefore, after drinking coffee whether changing of amniotic fluid index (AFI) and fetal renal artery blood flow (FRABF, RI, Resistive index; PI, Pulsatility index) were evaluated in this study.

**Methods:**

This clinical study was performed with two groups. For the study group, 63 participants with isolated borderline oligohydramnios who agreed to drink one cup of instant coffee were included in this study while 63 participants with isolated borderline oligohydramnios who did not drink one cup of instant coffee formed the control group. AFI, RI and PI were evaluated both before and after coffee intake.

**Results:**

Maternal characteristics of all study population were homogenous. FRABF indices were similar in both before and after coffee consumption. AFI was increased significantly six hours after drinking coffee (p<0.001).

**Conclusions:**

The coffee consumption increased the amniotic fluid volume. However it does not seem to affect on FRABF. According to our study findings, coffee consumption may offer a new opportunity to improve amniotic fluid volume for pregnant women with oligohydramnios.

## INTRODUCTION

Amniotic fluid is necessary for fetal comfort, growth, and development.^1^ A major source of amniotic fluid is fetal urine (800-1200 mL/day) and fetal lung fluid (170 mL/day) in the third trimester.^2^,^3^ Coffee, tea, and cocoa include the plant alkaloid caffeine. They are frequently (one or two cups /day) consumed throughout pregnancy. Caffeine metabolism slows down during pregnancy and has a longer half-life (1.5 to 3.5 times) than in non-pregnant women.^4^ Caffeine passes through the placenta and is detectable in the amniotic fluid, fetal urine, and fetal plasma.^5^

The American College of Obstetricians and Gynecologists has stated that a sensible amount (<200 mg/day) of caffeine consumption for pregnant women does not appear to have a favorable efficacy on birth weight.^6^ The Food and Drug Administration (FDA) reports one cup of instant coffee contains 93 mg of caffeine. Although there are a few studies evaluating caffeine effects on pregnancy, a diuretic effect of caffeine on fetal kidneys has not been reported. Hence, in the present study, we examined the effect of coffee consumption on fetal renal artery blood flow and AFI in the third trimester of pregnancy.

## METHODS

This clinical study was performed in The Clinic of Obstetrics of Health Sciences University, Kayseri City Hospital, Kayseri, Turkey. We arranged this study according to Declaration of Helsinki and received approval from the Ethics Committee of Erciyes University (2017/489). We also received informed consent from all participants while registered through ClinicalTrials.gov Identifier: NCT03798275.

Pregnant women with no perinatal risk in middle of third trimester were included in this study in the course of routine antenatal examinations between January 2018 and August 2018. For the study population, we preferred and randomized patients with isolated borderline oligohydramnios to examine the effect of one cup of instant coffee (2 g Nescafe Clasico sachets contains nearly 65 mg caffeine) consumption on AFI and blood flow of fetal kidney. Also, the control group for this study (age matched healthy pregnant women with isolated borderline oligohydramnios who did not want to drink coffee) was formed to compare the effect of caffeine.^7^

Exclusion criteria of our study were multiple pregnancy, polyhydramnios, any consumption of nutrient or fluid within the 4 hours previous to ultrasonographic examination,^8^ obesity, diabetes, hypertension, or preeclampsia, placental localization anomalies, ruptured fetal membranes, fetal anomaly and growth restriction. Also, pregnant women consuming coffee within the last seven days previous to ultrasonographic examination were excluded.

To eliminate inter-observer variability, all study patients were evaluated by the same specialist (EA) with the same ultrasound device (Toshiba Xario 200 3.5MHz). The sonographer was blinded to the intervention. Amniotic fluid was calculated according to the amniotic fluid index (AFI) method. After the first ultrasonographic evaluation, pregnant women with an AFI of 5.1-8 cm were included in the study group (borderline oligohydramnios).^9^ All ultrasonographic parameters were measured in a single position of the patient (flat supine position) without fetal breathing and fetal movement and measured trans-abdominally from the coronal plane of the fetal abdomen to provide optimal images of aorta, kidneys, and renal arteries.^10^ Fetal renal artery blood flow was evaluated at the initial level of the left renal artery originating from the abdominal aorta and three consecutive measurements were recorded as averages.^10^

Caffeine taken orally reaches its peak blood plasma levels in 15 to 45 minutes and the approximate half-life ranges from 5 to 6 hours.^11^ Therefore, volunteer pregnant women with borderline oligohydramnios who agreed drinking coffee were re-evaluated between the fourth and sixth hours of coffee intake. Taking any nutrients or liquid was not permitted until the ultrasonographic evaluation was repeated after coffee consumption. First (before coffee consumption) and second (after coffee consumption) measurements such as fetal renal artery Doppler [resistive index (RI) and pulsatility index (PI)] and AFI were compared. Pregnant women belong to the control group were re-evaluated after four to six hours of starvation. First and second measurements were compared. After evaluation, all study patients were monitored and managed according to modern obstetric guidelines.

The Shapiro-Wilk test was used to test the normality assumption of the data, and the Levene test was used to test the variance homogeneity assumption. Parametric comparisons were made using a t-test, and non-parametric comparisons were made using the Mann–Whitney U test. The comparisons of more than two groups were investigated using ANOVA followed by Tukey’s post hoc test. For all comparisons, Minitab 16 (Minitab Inc.; State College, PA, USA) was used. The difference between groups was considered statistically significant when the p < 0.05. All measurement values are expressed as mean ± SD and 95% confidence interval (CI).

## RESULTS

Sixty three patients who met the study criteria for the study group were included in this study. These patients were compared as a control group with 63 healthy pregnant women who randomized for study. Demographic characteristics of all study patients are shown in Table-I. Maternal age, body mass index (BMI), gestational age and gravidity were similar between groups.

**Table-I:** Maternal characteristics.

	Study group (n: 63)	Control group (n: 63)	P value
Maternal age (year)	25.14±5.29	25.70±2.92	0.547
Gravidity	2.07±1.01	2.14±1.04	0.753
Gestational age at ultrasound examination (week)	36.16±0.68	36.44±0.54	0.058
BMI (kg/m^2^)	26.90±1.25	27.05±1.41	0.944

Values are expressed as mean ± SD. BMI, body mass index.

Different superscript letters indicate statistically significant differences. All measurement values are expressed as mean ±SD and 95% confidence interval (CI). RI, resistive index; PI, pulsatility index.

**Fig.1:**
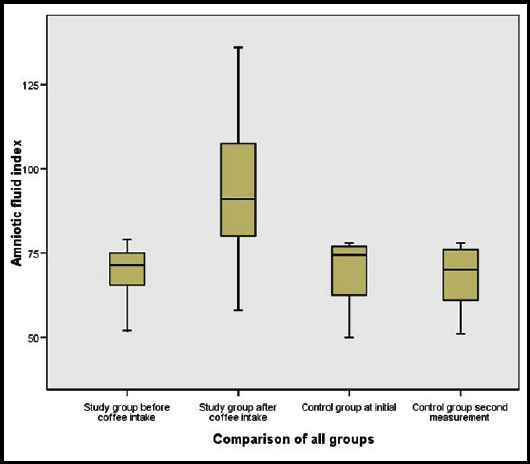
The box-plot graphic of the effect of coffee consumption on amniotic fluid index (mm).

All groups were evaluated two times. The comparison of ultrasonographic parameters between groups is shown in Table-II.. Fetal renal artery RI values and fetal renal artery PI values were similar among groups. AFI was significantly increased in the study group after drinking coffee (p<0.001). However, AFI was similar among groups except in the study group post coffee. The mean AFI values in the study population before coffee consumption and after coffee consumption, and in the control group at initial and after starvation were evaluated and compared and showed in Fig.1 as box plot graphic.

**Table-II:** Comparison of ultrasonographic parameters.

	Study group before coffee intake (n: 63)	Study group after coffee intake (n: 63)	Control group initial measurements (n: 63)	Control group after starvation (n: 63)	P value
Amniotic fluid index (mm)	68.77±8.3^a^ (66.23-71.32)	92.8±18.9^b^ (87.02-98.57)	69.44±9.1^a^ (65.05-70.43)	67.74±8.8^a^ (65.05-70.43)	<0.001
Fetal renal artery RI	0.85±0.08 (0.82-0.88) (2.07-2.36)	0.86±0.09 (0.83-0.90)	0.84±0.07 (0.82-0.88)	0.83±0.09 (0.81-0.87)	0.874
Fetal renal artery PI	2.21±0.47	2.15±0.53 (1.98-2.31)	2.16±0.53 (2.00-2.33)	2.12±0.52 (2.00-2.32)	0.947

Different superscript letters indicate statistically significant differences. All measurement values are expressed as mean ±SD and 95% confidence interval (CI). RI, resistive index; PI, pulsatility index.

## DISCUSSION

In this study, we examined the effect of one cup of instant coffee intake on AFV and blood flow of fetal kidney in the middle of the third trimester of pregnancy with isolated borderline oligohydramnios. It is known that there are some adverse perinatal outcomes may occur in pregnant women with borderline oligohydramnios.^10^ In terms of demographic characteristics, all study population seemed homogenous (Table-I). We assessed pregnant women between the 34th and 37th weeks of gestation (late preterm) to increase gestational time by treating decreased amniotic fluid, and to increase the term delivery rate, because there are many adverse neonatal outcomes in this period (such as respiratory distress syndrome, prolonged stay in neonatal intensive care unite, neonatal jaundice, transient tachypnea of the newborn).^12^

There are many studies investigating the effect of coffee consumption. One cup of coffee consumption seems to be safe and provides a clinically usable nutrient to increase AFV in the third trimester of pregnancy. Although coffee contains caffeine, clinical studies have not shown any clear association with adverse pregnancy outcomes.^13^-^16^ However, some authors report that high caffeine intake (for intake of 200 or more mg/day) during pregnancy may lead to increased risk of spontaneous abortion,^17^,^18^ fetal growth restriction,^19^ and preterm delivery.^20^ Although there are many investigations on coffee consumption during pregnancy, the effect of the recommended dose of coffee consumption on fetal renal blood flow and AFI has not been investigated. Therefore, this is the first study evaluating the effect of coffee consumption on fetal renal artery blood flow and AFV in the third trimester of pregnancy.

According to our study findings, fetal renal artery blood flow indices (both RI and PI) were not significantly changed owing to coffee intake. However, AFI was significantly increased six hours after coffee consumption (Fig.1). The increase in the AFV may occur owing to the diuretic effect of caffeine. Methylxanthines such as caffeine can restrict activity of phosphodiesterase in the proximal tubule of the fetal kidney, which may lead to a diuretic effect.^21^ Caffeine does not augment the glomerular filtration ratio of the kidneys,^22^ but the diuretic influence may be associated with the natriuretic influence following adenosine receptor inhibition. Thus, it increases dissolved substances and water excretion.

Currently, there is no effective long term treatment for oligohydramnios, but there is short-term treatment such as oral rehydration therapy (ORT) to improve AFV and to prolong gestation for fetal well-being. If delivery for pregnant women with oligohydramnios is not indicated or gestational age of these patients is less than the 37^th^ week, ORT may have some benefit. However, treatments such as amnioinfusion or intravenous fluid administration are both more difficult and more uncertain than ORT. After hydration therapy, maternal sodium concentration and plasma osmolality decrease. This causes water flow from mother to her fetus; it also improves uteroplacental perfusion.^23^ In these patients, one cup of coffee consumption and addition maternal ORT seems to be an easy, non-invasive, and clinically usable method to increase AFI to prevent the adverse effects of oligohydramnios.

### Limitation of the Study

The study population was not large enough. Another limitation is that we could not evaluate effects of coffee on pregnant women with normal AFV. One of these reasons is that many pregnant women with normal AFV did not agree to drink coffee. Another reason is that it is not needed treatment to improve the AFV. Similar publications are taken as examples.^7^ It would be better if we measured AFI a few more times after intervention to evaluate long term effect of coffee and.

## CONCLUSION

We found that the coffee consumption increased the AFV. However it does not seem to affect on fetal renal artery blood flow. According to our study findings, coffee consumption may offer a new opportunity to improve AFV for pregnant women with isolated borderline oligohydramnios. Further new clinical studies to evaluate both AFV and late pregnancy outcome are needed.

### Authors’ Contribution

**ICM&YM** conceived, designed and did statistical analysis & editing of manuscript.

**EA&MES** did data collection and manuscript writing.

**ICM&YM** did review, final approval of manuscript and is responsible for integrity of research.
